# Supplementary material for: Meiotic gene silencing complex MTREC/NURS recruits the nuclear exosome to YTH-RNA-binding protein Mmi1
Source: PLoS Genet. 2020 Feb 3;16(2):e1008598. doi: 10.1371/journal.pgen.1008598 (PMC7018101; doi:10.1371/journal.pgen.1008598)
Supplement: S1 Fig — (A) Localization of Rrp6, Red1 and Mmi1 in wild-type, red1Δ, mei4Δ, mmi1Δ mei4Δ, and pab2Δ cells. Cells expressing Rrp6-YFP (green), Red1-mCherry (red) or CFP-Mmi1 (blue) from the respective endogenous loci were observed during exponential growth in YE liquid medium. Dotted lines indicate the shape of cells. Boxed regions are magnified in Fig 1A. (B) Localization of Dis3 and Rrp4 in wild-type and red1Δ cells. red1Δ cells expressing Dis3-GFP or Rrp4-GFP from the respective endogenous loci were observed. Boxed regions are magnified in Fig 1B. (C) Localization of Pla1, Red1 and Mmi1 in wild-type and red1Δ cells. Cells expressing Pla1-YFP (green), Red1-mCherry (red) and CFP-Mmi1 (blue) were examined. Boxed regions are magnified in Fig 1C. (D) Localization of Pab2, Red1, and Mmi1 in wild-type and red1Δ cells. Cells expressing Pab2-YFP (green), Red1-mCherry (red) and CFP-Mmi1 (blue) were examined. Boxed regions are magnified in Fig 1D. Scale bars: 5 μm. (PDF) [file pgen.1008598.s001.pdf]

S1 Fig.

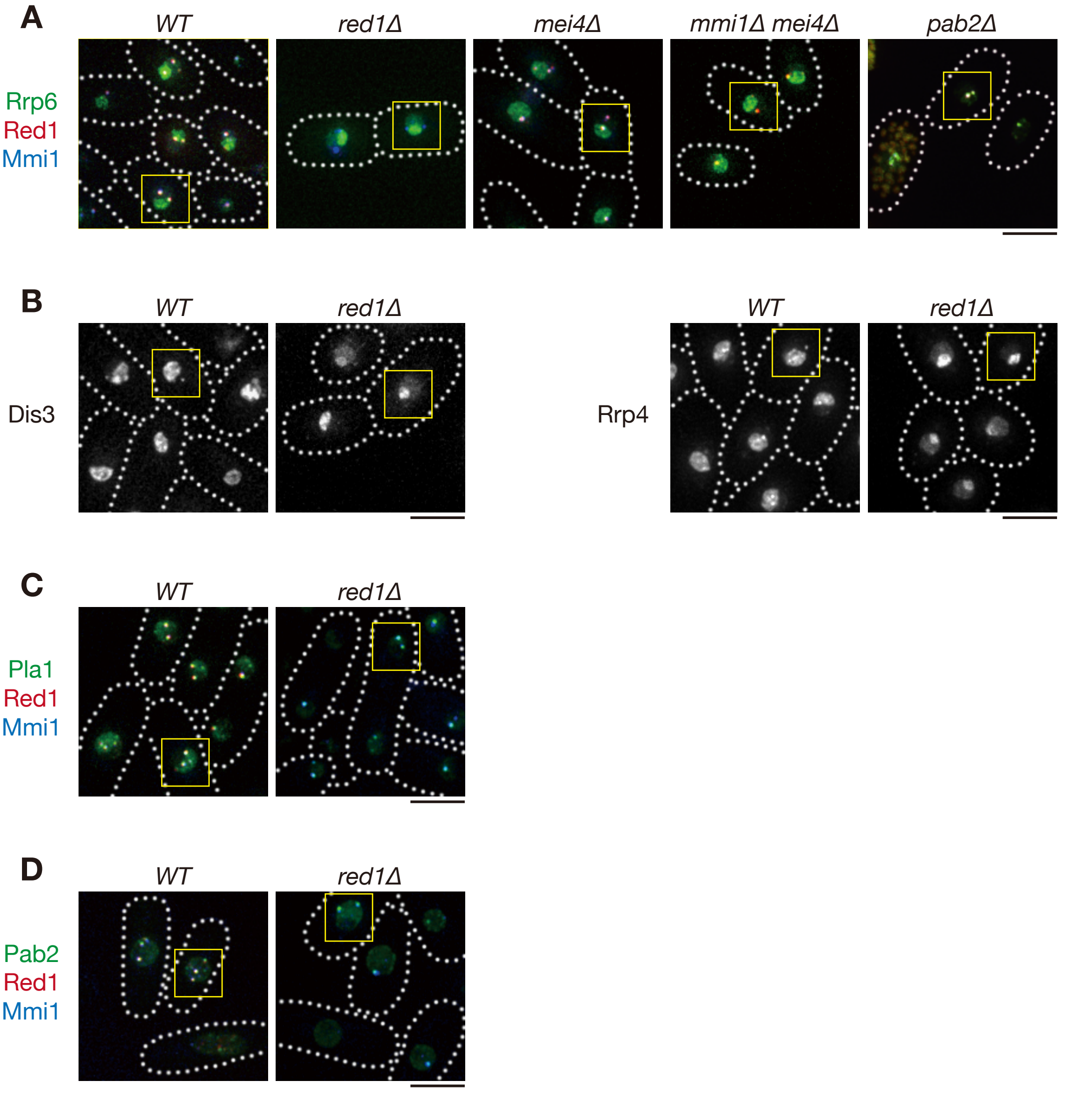

S1 Fig. Red1 is required for nuclear foci formation of exosome.

(**A**) Localization of Rrp6, Red1 and Mmi1 in wild-type, *red1Δ*, *mei4Δ*, *mmi1Δ mei4Δ*, and *pab2Δ* cells. Cells expressing Rrp6-YFP (green), Red1-mCherry (red) or CFP-Mmi1 (blue) from the respective endogenous loci were observed during exponential growth in YE liquid medium. Dotted lines indicate the shape of cells. Boxed regions are magnified in Fig 1A.

(**B**) Localization of Dis3 and Rrp4 in wild-type and *red1Δ* cells. *red1Δ* cells expressing Dis3-GFP or Rrp4-GFP from the respective endogenous loci were observed. Boxed regions are magnified in Fig 1B.

(**C**) Localization of Pla1, Red1 and Mmi1 in wild-type and *red1Δ* cells. Cells expressing Pla1-YFP (green), Red1-mCherry (red) and CFP-Mmi1 (blue) were examined. Boxed regions are magnified in Fig 1C.

(**D**) Localization of Pab2, Red1, and Mmi1 in wild-type and *red1Δ* cells. Cells expressing Pab2-YFP (green), Red1-mCherry (red) and CFP-Mmi1 (blue) were examined. Boxed regions are magnified in Fig 1D. Scale bars: 5 μm.
