# Supplementary material for: Meiotic gene silencing complex MTREC/NURS recruits the nuclear exosome to YTH-RNA-binding protein Mmi1
Source: PLoS Genet. 2020 Feb 3;16(2):e1008598. doi: 10.1371/journal.pgen.1008598 (PMC7018101; doi:10.1371/journal.pgen.1008598)
Supplement: S2 Fig — (A) Expression levels of truncated Red1 proteins. Cell extracts were prepared from exponentially growing cells expressing wild-type or truncated Red1-YFP in liquid YE medium and immunoblotted with anti-GFP antibody. γ-tubulin was used as a loading control. The asterisks indicate non-specific bands. (B) Localization of Rrp6 and Red1 in red1(Δ196–245) cells. red1(Δ196–245) cells expressing Rrp6-YFP (green) and Red1-mCherry (magenta) from the respective endogenous loci were observed. Dotted lines indicate the shape of cells. Boxed region is magnified in Fig 2C. Scale bar: 5 μm. (C) Localization of Rrp6, Red1 and Mmi1 in iss10Δ cells. iss10Δ cells expressing Rrp6-YFP (green), Red1-mCherry (red) and CFP-Mmi1 (blue) were examined. Boxed region is magnified in Fig 2D. Scale bar: 5 μm. (PDF) [file pgen.1008598.s002.pdf]

S2 Fig.

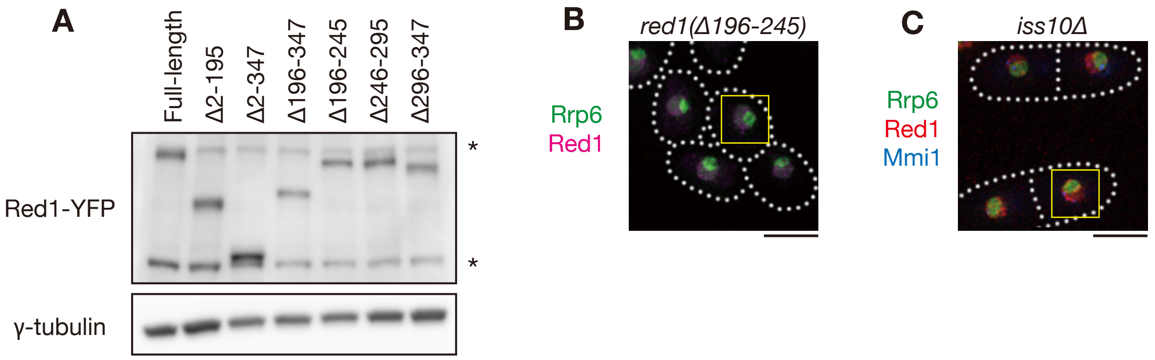

S2 Fig. Red1(Δ196-245) is defective in Rrp6 foci formation.

(**A**) Expression levels of truncated Red1 proteins. Cell extracts were prepared from exponentially growing cells expressing wild-type or truncated Red1-YFP in liquid YE medium and immunoblotted with anti-GFP antibody. γ-tubulin was used as a loading control. The asterisks indicate non-specific bands.

(**B**) Localization of Rrp6 and Red1 in *red1(Δ196-245)* cells. *red1(Δ196-245)* cells expressing Rrp6-YFP (green) and Red1-mCherry (magenta) from the respective endogenous loci were observed. Dotted lines indicate the shape of cells. Boxed region is magnified in Fig 2C. Scale bar: 5 μm.

(**C**) Localization of Rrp6, Red1 and Mmi1 in *iss10Δ* cells. *iss10Δ* cells expressing Rrp6-YFP (green), Red1-mCherry (red) and CFP-Mmi1 (blue) were examined. Boxed region is magnified in Fig 2D. Scale bar: 5 μm.
