# Supplementary material for: Meiotic gene silencing complex MTREC/NURS recruits the nuclear exosome to YTH-RNA-binding protein Mmi1
Source: PLoS Genet. 2020 Feb 3;16(2):e1008598. doi: 10.1371/journal.pgen.1008598 (PMC7018101; doi:10.1371/journal.pgen.1008598)
Supplement: S3 Fig — (A) Growth profiles of wild-type (red1-YFP), red1Δ, iss10Δ and red1(Δ196–245) cells. Ten-fold serial dilutions of cells were spotted on YE medium and incubated at the indicated temperatures. (B) Expression of rec8 mRNA and spo5 mRNA in wild-type (red1-YFP), red1Δ, iss10Δ and red1(Δ196–245) cells. Transcripts were quantified by RT-qPCR and normalized to act1. Error bars represent standard error of three independent samples. *P < 0.05; ***P < 0.001 compared with the wild-type red1-YFP strain (Student’s t-test). (C) Expression of ssm4 mRNA, rec8 mRNA, spo5 mRNA, cti6 PROMPT and rpl402 PROMPT in wild-type, mmi1Δ mei4Δ, red1Δ and rrp6Δ cells. Transcripts were quantified by RT-qPCR and normalized to act1. Error bars represent standard error of three independent samples. *P < 0.05; **P < 0.01; ***P < 0.001 compared with the wild-type strain (Student’s t-test). (D) Expression of cti6 PROMPT and rpl402 PROMPT in wild-type (red1-YFP), red1Δ, red1(Δ196–245), and iss10Δ cells. Transcripts were quantified by RT-qPCR and normalized to act1. Error bars represent standard error of three independent samples. *P < 0.05; **P < 0.01; ***P < 0.001 compared with the wild-type red1-YFP strain (Student’s t-test). (PDF) [file pgen.1008598.s003.pdf]

S3 Fig.

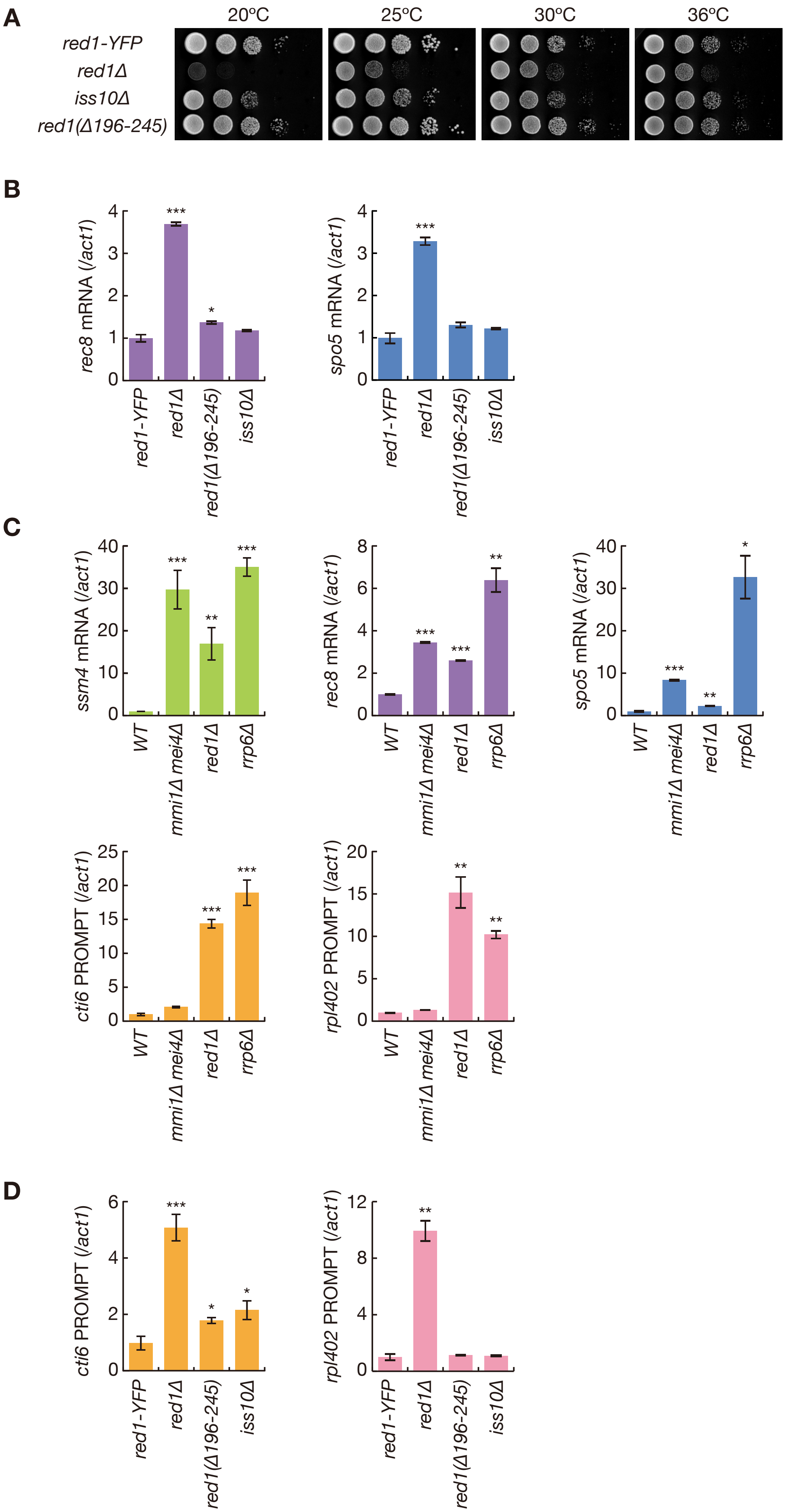
A
20°C
25°C
30°C
36°C
red1-YFP
red1Δ
iss10Δ
red1(Δ196-245)
B
rec8 mRNA (/act1)
spo5 mRNA (/act1)
red1-YFP
red1Δ
red1(Δ196-245)
iss10Δ
C
ssm4 mRNA (/act1)
rec8 mRNA (/act1)
spo5 mRNA (/act1)
cti6 PROMPT (/act1)
rpl402 PROMPT (/act1)
WT
mmi1Δ mei4Δ
red1Δ
rrp6Δ
D
cti6 PROMPT (/act1)
rpl402 PROMPT (/act1)
red1-YFP
red1Δ
red1(Δ196-245)
iss10Δ

S3 Fig. Red1(Δ196-245) is defective in meiotic transcript degradation.

(**A**) Growth profiles of wild-type (*red1-YFP*), *red1Δ*, *iss10Δ* and *red1(Δ196-245)* cells. Ten-fold serial dilutions of cells were spotted on YE medium and incubated at the indicated temperatures.

(**B**) Expression of *rec8* mRNA and *spo5* mRNA in wild-type (*red1-YFP*), *red1Δ*, *iss10Δ* and *red1(Δ196-245)* cells. Transcripts were quantified by RT-qPCR and normalized to *act1*. Error bars represent standard error of three independent samples. *$P < 0.05$; ***$P < 0.001$ compared with the wild-type *red1-YFP* strain (Student's *t*-test).

(**C**) Expression of *ssm4* mRNA, *rec8* mRNA, *spo5* mRNA, *cti6* PROMPT and *rpl402* PROMPT in wild-type, *mmi1Δ mei4Δ*, *red1Δ* and *rrp6Δ* cells. Transcripts were quantified by RT-qPCR and normalized to *act1*. Error bars represent standard error of three independent samples. *$P < 0.05$; **$P < 0.01$; ***$P < 0.001$ compared with the wild-type strain (Student's *t*-test).

(**D**) Expression of *cti6* PROMPT and *rpl402* PROMPT in wild-type (*red1-YFP*), *red1Δ*, *red1(Δ196-245)*, and *iss10Δ* cells. Transcripts were quantified by RT-qPCR and normalized to *act1*. Error bars represent standard error of three independent samples. *$P < 0.05$; **$P < 0.01$; ***$P < 0.001$ compared with the wild-type *red1-YFP* strain (Student's *t*-test).
