# Supplementary material for: Meiotic gene silencing complex MTREC/NURS recruits the nuclear exosome to YTH-RNA-binding protein Mmi1
Source: PLoS Genet. 2020 Feb 3;16(2):e1008598. doi: 10.1371/journal.pgen.1008598 (PMC7018101; doi:10.1371/journal.pgen.1008598)
Supplement: S4 Fig — (A) Co-immunoprecipitation of Rrp6 and Mmi1 in wild-type cells. Native cell extracts were prepared from exponentially growing cells expressing Rrp6-3HA from a plasmid in MM medium and subjected to immunoprecipitation with anti-HA antibody. Cells carrying an empty vector were used as a negative control. Precipitates and 10% total cell extracts were then immunoblotted with anti-Mmi1 and anti-HA antibodies. (B) The effect of RNase treatment on interaction between Rrp6 and Mmi1. Native cell extracts were incubated with or without RNase and subjected to immunoprecipitation with anti-HA antibody. (PDF) [file pgen.1008598.s004.pdf]

S4 Fig.

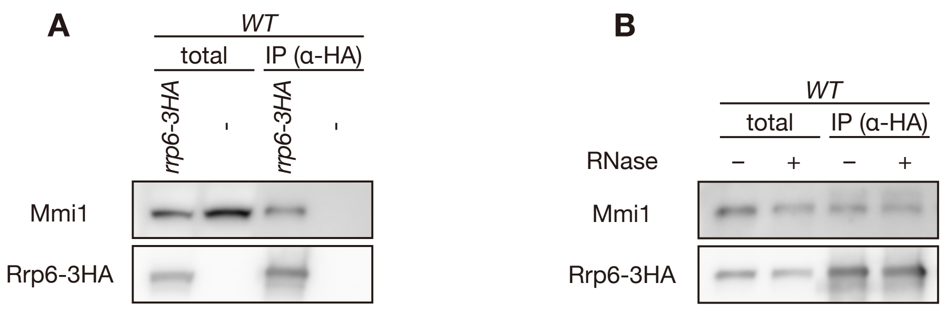

S4 Fig. Rrp6 physically interacts with Mmi1.

(**A**) Co-immunoprecipitation of Rrp6 and Mmi1 in wild-type cells. Native cell extracts were prepared from exponentially growing cells expressing Rrp6-3HA from a plasmid in MM medium and subjected to immunoprecipitation with anti-HA antibody. Cells carrying an empty vector were used as a negative control. Precipitates and 10% total cell extracts were then immunoblotted with anti-Mmi1 and anti-HA antibodies.

(**B**) The effect of RNase treatment on interaction between Rrp6 and Mmi1. Native cell extracts were incubated with or without RNase and subjected to immunoprecipitation with anti-HA antibody.
