# Supplementary material for: Meiotic gene silencing complex MTREC/NURS recruits the nuclear exosome to YTH-RNA-binding protein Mmi1
Source: PLoS Genet. 2020 Feb 3;16(2):e1008598. doi: 10.1371/journal.pgen.1008598 (PMC7018101; doi:10.1371/journal.pgen.1008598)
Supplement: S5 Fig — (A) Growth profiles of rrp6Δ cells expressing Red1, Rrp6-GFP, Mmi1, or chimeric proteins composed of Rrp6, GFP, and full-length or truncated Mmi1 from plasmids. Ten-fold serial dilutions of cells were spotted on MM medium and incubated at the indicated temperatures. (B) Expression of mei4 mRNA, ssm4 mRNA, and cti6 PROMPT in rrp6Δ cells expressing Red1, Rrp6-GFP, Mmi1, or chimeric proteins composed of Rrp6, GFP, and full-length or truncated Mmi1 from plasmids. Transcripts were quantified by RT-qPCR and normalized to act1. Error bars represent standard error of three independent samples. **P < 0.01; ***P < 0.001 compared with cells carrying empty vector (Student’s t-test). (C) Growth profiles of mmi1-ts3 cells expressing Red1, Rrp6-GFP, Mmi1, or chimeric proteins composed of Rrp6, GFP, and full-length or truncated Mmi1 from plasmids. Ten-fold serial dilutions of cells were spotted on MM medium and incubated at the indicated temperatures. (D) Expression of mei4 mRNA and ssm4 mRNA in mmi1-ts3 cells expressing Red1, Rrp6-GFP, Mmi1, or chimeric proteins composed of Rrp6, GFP, and full-length or truncated Mmi1 from plasmids. Cells were grown in liquid MM medium at 25°C and shifted to 37°C for 4 hours. Transcripts were quantified by RT-qPCR and normalized to act1. Error bars represent standard error of three independent samples. **P < 0.01; ***P < 0.001 compared with cells carrying empty vector at 37˚C (Student’s t-test). (PDF) [file pgen.1008598.s005.pdf]

S5 Fig.

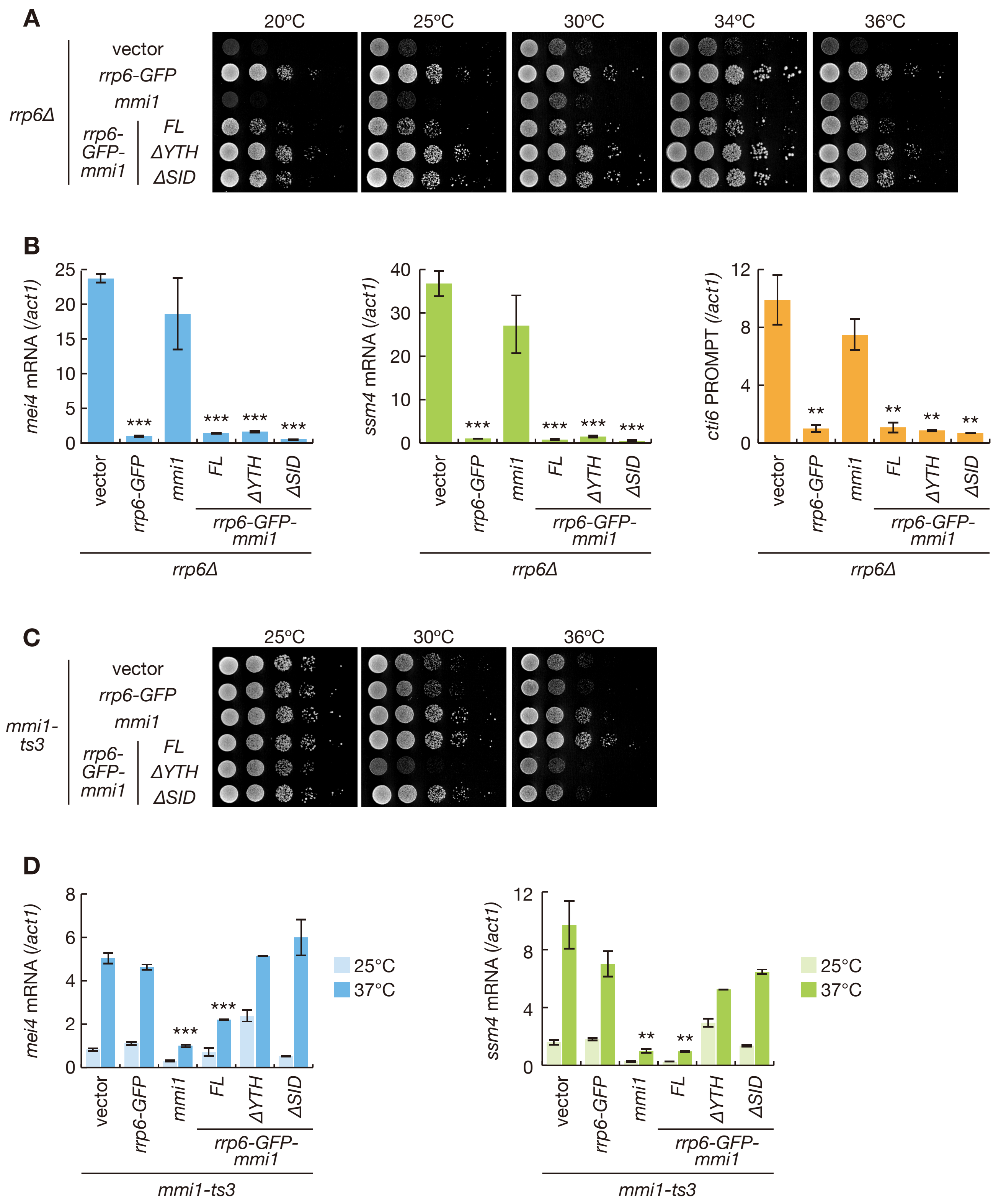
A
20°C
25°C
30°C
34°C
36°C
vector
rrp6-GFP
mmi1
rrp6Δ
rrp6-GFP-mmi1
FL
ΔYTH
ΔSID
B
mei4 mRNA (/act1)
ssm4 mRNA (/act1)
cti6 PROMPT (/act1)
***
**
C
mmi1-ts3
D
25°C
37°C

S5 Fig. Chimeric Rrp6-GFP-Mmi1 protein maintains the function of Rrp6 and Mmi1.

(**A**) Growth profiles of *rrp6Δ* cells expressing Red1, Rrp6-GFP, Mmi1, or chimeric proteins composed of Rrp6, GFP, and full-length or truncated Mmi1 from plasmids. Ten-fold serial dilutions of cells were spotted on MM medium and incubated at the indicated temperatures.

(**B**) Expression of *mei4* mRNA, *ssm4* mRNA, and *cti6* PROMPT in *rrp6Δ* cells expressing Red1, Rrp6-GFP, Mmi1, or chimeric proteins composed of Rrp6, GFP, and full-length or truncated Mmi1 from plasmids. Transcripts were quantified by RT-qPCR and normalized to *act1*. Error bars represent standard error of three independent samples. **$P < 0.01$; ***$P < 0.001$ compared with cells carrying empty vector (Student's *t*-test).

(**C**) Growth profiles of *mmi1-ts3* cells expressing Red1, Rrp6-GFP, Mmi1, or chimeric proteins composed of Rrp6, GFP, and full-length or truncated Mmi1 from plasmids. Ten-fold serial dilutions of cells were spotted on MM medium and incubated at the indicated temperatures.

(**D**) Expression of *mei4* mRNA and *ssm4* mRNA in *mmi1-ts3* cells expressing Red1, Rrp6-GFP, Mmi1, or chimeric proteins composed of Rrp6, GFP, and full-length or truncated Mmi1 from plasmids. Cells were grown in liquid MM medium at 25°C and shifted to 37°C for 4 hours. Transcripts were quantified by RT-qPCR and normalized to *act1*. Error bars represent standard error of three independent samples. **$P < 0.01$; ***$P < 0.001$ compared with cells carrying empty vector at 37˚C (Student's *t*-test).
