# Supplementary material for: Meiotic gene silencing complex MTREC/NURS recruits the nuclear exosome to YTH-RNA-binding protein Mmi1
Source: PLoS Genet. 2020 Feb 3;16(2):e1008598. doi: 10.1371/journal.pgen.1008598 (PMC7018101; doi:10.1371/journal.pgen.1008598)
Supplement: S6 Fig — (A) Expression of rec8 mRNA, spo5 mRNA, and rpl402 PROMPT in red1Δ cells expressing Red1, Rrp6-GFP, Mmi1 or chimeric proteins composed of Rrp6, GFP, and full-length or truncated Mmi1 from plasmids in liquid MM medium. Transcripts of each gene were analyzed by RT-qPCR and normalized to act1. Error bars represent standard error of three independent samples. *P < 0.05; **P < 0.01 compared with cells carrying empty vector (Student’s t-test). (B) Growth profiles of red1Δ cells expressing Red1, Rrp6-GFP, Mmi1, or chimeric proteins composed of Rrp6, GFP, and full-length or truncated Mmi1 from plasmids. Ten-fold serial dilutions of cells were spotted on MM medium and incubated at the indicated temperatures. (C) Expression levels of chimeric Rrp6-YFP-Mmi1 proteins. Cell extracts were prepared from exponentially growing cells expressing Rrp6-YFP or chimeric proteins composed of Rrp6, YFP, and full-length or truncated Mmi1 from plasmids in liquid MM medium and immunoblotted with anti-GFP antibody. γ-tubulin was used as a loading control. (D) Expression of rec8 mRNA, spo5 mRNA and rpl402 PROMPT in red1Δ mmi1Δ mei4Δ cells expressing Red1, Rrp6-GFP, Mmi1, or chimeric proteins composed of Rrp6, GFP, and full-length or truncated Mmi1 from plasmids in liquid MM medium. Transcripts were analyzed by RT-qPCR and normalized to act1. Error bars represent standard error of three independent samples. *P < 0.05; **P < 0.01; ***P < 0.001 compared with cells carrying empty vector (Student’s t-test). (E) Co-immunoprecipitation of endogenous Mmi1 and chimeric Rrp6-GFP-Mmi1 proteins. Native cell extracts were prepared from exponentially growing cells expressing chimeric proteins composed of Rrp6, GFP, and full-length or truncated Mmi1 from plasmids in liquid MM medium and subjected to immunoprecipitation with anti-GFP antibody. Precipitates and 10% total cell extracts were then immunoblotted with anti-Mmi1 and anti-GFP antibodies. (PDF) [file pgen.1008598.s006.pdf]

S6 Fig.

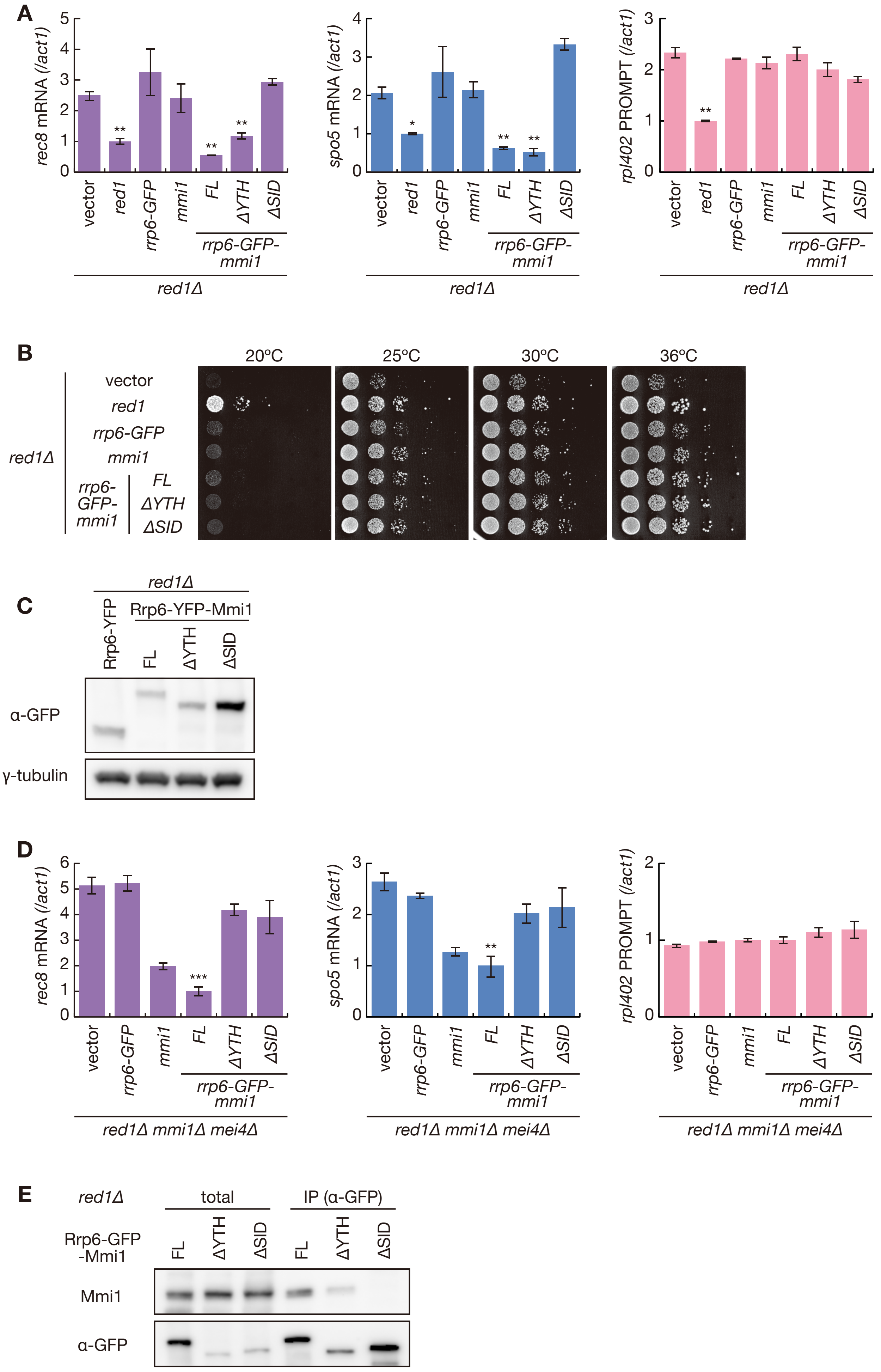
A
rec8 mRNA (/act1)
spo5 mRNA (/act1)
rpl402 PROMPT (/act1)
vector
red1
rrp6-GFP
mmi1
FL
ΔYTH
ΔSID
rrp6-GFP-mmi1
red1Δ
B
20°C
25°C
30°C
36°C
C
Rrp6-YFP
Rrp6-YFP-Mmi1
α-GFP
γ-tubulin
D
red1Δ mmi1Δ mei4Δ
E
total
IP (α-GFP)
Rrp6-GFP-Mmi1
Mmi1

S6 Fig. Interaction between Rrp6 and Mmi1 is essential for meiotic transcript elimination.

(**A**) Expression of *rec8* mRNA, *spo5* mRNA, and *rpl402* PROMPT in *red1Δ* cells expressing Red1, Rrp6-GFP, Mmi1 or chimeric proteins composed of Rrp6, GFP, and full-length or truncated Mmi1 from plasmids in liquid MM medium. Transcripts of each gene were analyzed by RT-qPCR and normalized to *act1*. Error bars represent standard error of three independent samples. *$P < 0.05$; **$P < 0.01$ compared with cells carrying empty vector (Student's *t*-test).

(**B**) Growth profiles of red1Δ cells expressing Red1, Rrp6-GFP, Mmi1, or chimeric proteins composed of Rrp6, GFP, and full-length or truncated Mmi1 from plasmids. Ten-fold serial dilutions of cells were spotted on MM medium and incubated at the indicated temperatures.

(**C**) Expression levels of chimeric Rrp6-YFP-Mmi1 proteins. Cell extracts were prepared from exponentially growing cells expressing Rrp6-YFP or chimeric proteins composed of Rrp6, YFP, and full-length or truncated Mmi1 from plasmids in liquid MM medium and immunoblotted with anti-GFP antibody. γ-tubulin was used as a loading control.

(**D**) Expression of *rec8* mRNA, *spo5* mRNA and *rpl402* PROMPT in *red1Δ mmi1Δ mei4Δ* cells expressing Red1, Rrp6-GFP, Mmi1, or chimeric proteins composed of Rrp6, GFP, and full-length or truncated Mmi1 from plasmids in liquid MM medium. Transcripts were analyzed by RT-qPCR and normalized to act1. Error bars represent standard error of three independent samples. *$P < 0.05$; **$P < 0.01$; ***$P < 0.001$ compared with cells carrying empty vector (Student's *t*-test).

(**E**) Co-immunoprecipitation of endogenous Mmi1 and chimeric Rrp6-GFP-Mmi1 proteins. Native cell extracts were prepared from exponentially growing cells expressing chimeric proteins composed of Rrp6, GFP, and full-length or truncated Mmi1 from plasmids in liquid MM medium and subjected to immunoprecipitation with anti-GFP antibody. Precipitates and 10% total cell extracts were then immunoblotted with anti-Mmi1 and anti-GFP antibodies.
