# Supplementary material for: Meiotic gene silencing complex MTREC/NURS recruits the nuclear exosome to YTH-RNA-binding protein Mmi1
Source: PLoS Genet. 2020 Feb 3;16(2):e1008598. doi: 10.1371/journal.pgen.1008598 (PMC7018101; doi:10.1371/journal.pgen.1008598)
Supplement: S7 Fig — (A) Localization of Dis3 and Rrp4 in rrp6Δ cells. rrp6Δ cells expressing Dis3-GFP or Rrp4-GFP from the respective endogenous loci were observed during exponential growth in liquid YE medium. Dotted lines indicate the shape of cells. Boxed regions are magnified in Fig 5A. (B) Localization of Red1 and Mmi1 in rrp6Δ cells. rrp6Δ cells expressing Red1-YFP (green) and CFP-Mmi1 (magenta) were examined. Boxed region is magnified in Fig 5B. (C) Localization of Dis3 and Rrp4 in red1Δ cells expressing Rrp6-YFP, YFP-Mmi1, or chimeric proteins composed of Rrp6, YFP, and full-length or truncated Mmi1. Dis3-mCherry and Rrp4-mCherry were expressed from the respective endogenous loci (green) and YFP-containing chimeric proteins were expressed from plasmids (magenta) in liquid SD medium. Boxed regions are magnified in Fig 5C. Scale bars: 5 μm. (PDF) [file pgen.1008598.s007.pdf]

S7 Fig.

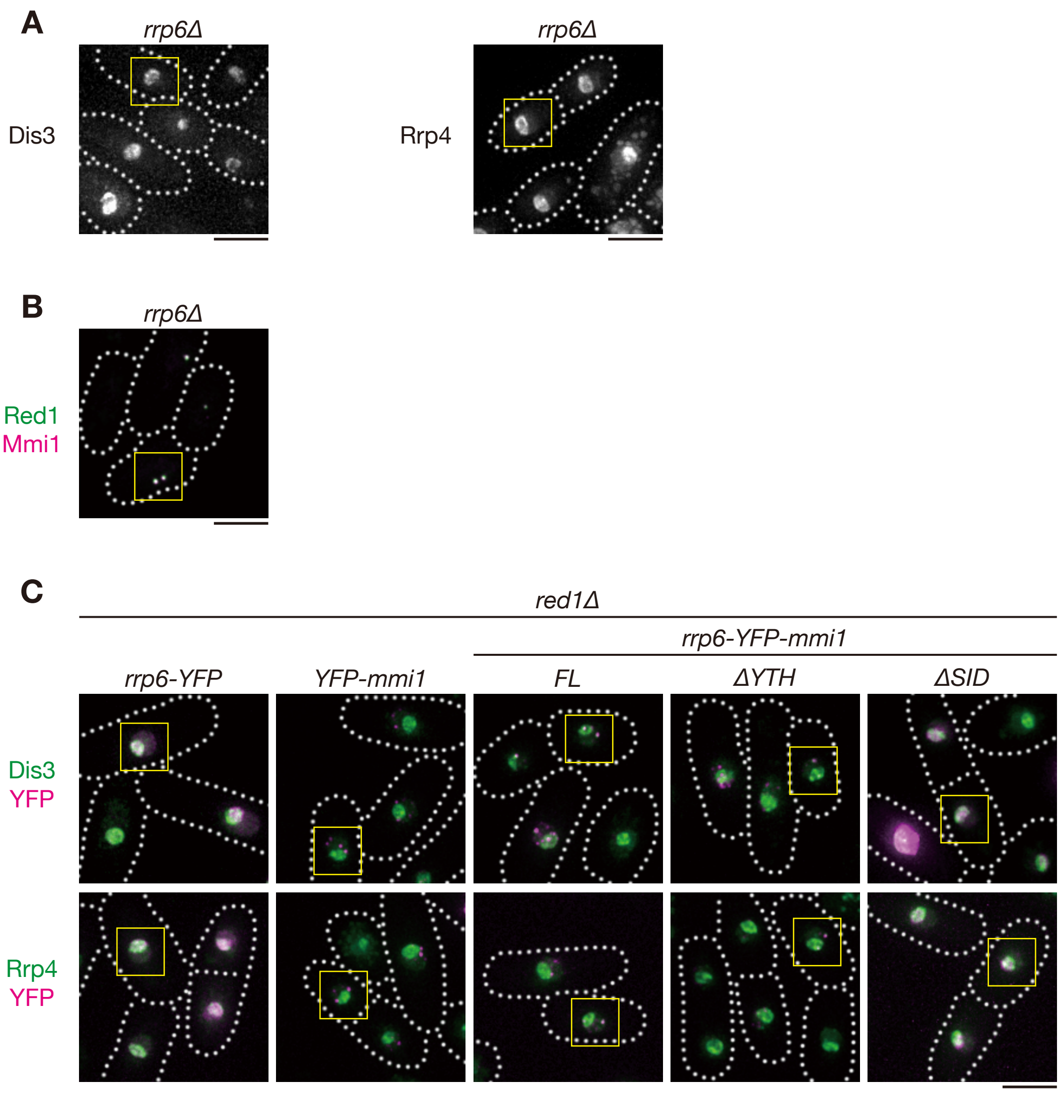

S7 Fig. Rrp6 is vital for nuclear foci formation of other exosome components.
(**A**) Localization of Dis3 and Rrp4 in *rrp6Δ* cells. *rrp6Δ* cells expressing Dis3-GFP or Rrp4-GFP from the respective endogenous loci were observed during exponential growth in liquid YE medium. Dotted lines indicate the shape of cells. Boxed regions are magnified in Fig 5A.
(**B**) Localization of Red1 and Mmi1 in *rrp6Δ* cells. *rrp6Δ* cells expressing Red1-YFP (green) and CFP-Mmi1 (magenta) were examined. Boxed region is magnified in Fig 5B.
(**C**) Localization of Dis3 and Rrp4 in *red1Δ* cells expressing Rrp6-YFP, YFP-Mmi1, or chimeric proteins composed of Rrp6, YFP, and full-length or truncated Mmi1. Dis3-mCherry and Rrp4-mCherry were expressed from the respective endogenous loci (green) and YFP-containing chimeric proteins were expressed from plasmids (magenta) in liquid SD medium. Boxed regions are magnified in Fig 5C. Scale bars: 5 μm.
