# Supplementary material for: Meiotic gene silencing complex MTREC/NURS recruits the nuclear exosome to YTH-RNA-binding protein Mmi1
Source: PLoS Genet. 2020 Feb 3;16(2):e1008598. doi: 10.1371/journal.pgen.1008598 (PMC7018101; doi:10.1371/journal.pgen.1008598)
Supplement: S8 Fig — Expression of rec8 mRNA and spo5 mRNA in mtl1-cs5 cells expressing Mtl1 and Rrp6-GFP-Mmi1 from plasmids. Cells were grown in liquid MM medium at 30°C and shifted to 20°C for 2 hours. Transcripts were quantified by RT-qPCR and normalized to act1. Error bars represent standard error of three independent samples. *P < 0.05; **P < 0.01 compared with cells carrying empty vector at 20˚C (Student’s t-test). (PDF) [file pgen.1008598.s008.pdf]

S8 Fig.

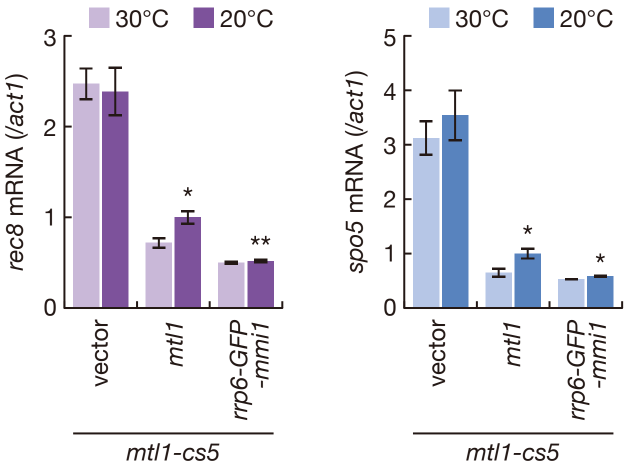

S8 Fig. Mtl1 mediates interaction between Rrp6 and Mmi1.

Expression of *rec8* mRNA and *spo5* mRNA in *mtl1-cs5* cells expressing Mtl1 and Rrp6-GFP-Mmi1 from plasmids. Cells were grown in liquid MM medium at 30°C and shifted to 20°C for 2 hours. Transcripts were quantified by RT-qPCR and normalized to *act1*. Error bars represent standard error of three independent samples. *$P < 0.05$; **$P < 0.01$ compared with cells carrying empty vector at 20˚C (Student's *t*-test).
