# Supplementary material for: Meiotic gene silencing complex MTREC/NURS recruits the nuclear exosome to YTH-RNA-binding protein Mmi1
Source: PLoS Genet. 2020 Feb 3;16(2):e1008598. doi: 10.1371/journal.pgen.1008598 (PMC7018101; doi:10.1371/journal.pgen.1008598)
Supplement: S1 Table — (PDF) [file pgen.1008598.s009.pdf]

S1 Table. Strains used in this study.

Strain name	Genotype	Figure	Derived from
JS59	*h90 ade6-M216 leu1 red1::bsdR*	2E, 4A, 4D, S3A, S3B, S3C, S3D, S6A, S6B, S6C, S6E	Ref. 33
JS96	*h90 ade6-M210 leu1 ura4-D18 mmi1::kanR mei4::ura4+*	S3C	Ref. 33
JS127	*h90 ade6-M210 leu1 rrp6-YFP-bsdR red1-mCherry-hphR natR-CFP-mmi1*	1A, S1A	This study
JS128	*h90 ade6-M216 leu1 ura4-D18 red1::bsdR rrp6-YFP-kanR LEU2-CFP-mmi1*	1A, S1A	This study
JS129	*h90 ade6-M216 leu1 mei4::ura4+ rrp6-YFP-bsdR red1-mCherry-hphR natR-CFP-mmi1*	1A, S1A	This study
JS130	*h90 ade6-M210 leu1 ura4-D18 mei4::ura4+ mmi1::kanR rrp6-YFP-bsdR red1-mCherry-hphR*	1A, S1A	This study
JS131	*h90 ade6-M216 leu1 pab2::kanR rrp6-YFP-bsdR red1-mCherry-hphR natR-CFP-mmi1*	1A, S1A	This study
JS132	*h90 ade6-M216 leu1 red1::bsdR dis3-GFP-kanR*	1B, S1B	This study
JS133	*h90 ade6-M216 leu1 red1::bsdR rrp4-GFP-kanR*	1B, S1B	This study
JS134	*h90 ade6-M216 leu1 pla1-YFP-kanR red1-mCherry-hphR natR-CFP-mmi1*	1C, S1C	This study
JS135	*h90 ade6-M216 leu1 red1::bsdR pla1-YFP-kanR natR-CFP-mmi1*	1C, S1C	This study
JS136	*h90 ade6-M216 leu1 pab2-YFP-kanR red1-mCherry-hphR natR-CFP-mmi1*	1D, S1D	This study
JS137	*h90 ade6-M216 leu1 red1::bsdR pab2-YFP-kanR natR-CFP-mmi1*	1D, S1D	This study
JS138	*h90 ade6-M216 leu1 red1-YFP-kanR*	2A, 2B, 2E, 3B, S2A, S3A, S3B, S3D	This study
JS139	*h90 ade6-M216 leu1 red1(Δ2-195)-YFP-kanR*	2A, 2B, S2A	This study
JS140	*h90 ade6-M216 leu1 red1(Δ2-347)-YFP-kanR*	2A, 2B, S2A	This study
JS143	*h90 ade6-M216 leu1 red1(Δ196-347)-YFP-kanR*	2A, 2B, S2A	This study
JS144	*h90 ade6-M216 leu1 red1(Δ196-245)-YFP-kanR*	2A, 2B, 2E, 3B, S2A, S3A, S3B, S3D	This study
JS145	*h90 ade6-M216 leu1 red1(Δ246-295)-YFP-kanR*	2A, 2B, S2A	This study
JS146	*h90 ade6-M216 leu1 red1(Δ296-347)-YFP-kanR*	2A, 2B, S2A	This study

JS149	*h90 ade6-M210 leu1 iss10::kanR rrp6-YFP-bsdR red1-mCherry-hphR natR-CFP-mmi1*	2D, S2C	This study
JS150	*h90 ade6-M216 leu1 ura4-D18 red1(d196-245)-mCherry-hphR rrp6-YFP-bsdR*	2C, S2B	This study
JS151	*h90 ade6-M210 leu1 rrp6::kanR*	S3C, S5A, S5B	This study
JS154	*h90 ade6-M216 leu1 rrp6::bsdR dis3-GFP-kanR*	5A, S7A	This study
JS155	*h90 ade6-M216 leu1 rrp6::bsdR rrp4-GFP-kanR*	5A, S7A	This study
JS156	*h90 ade6-M216 leu1 rrp6::bsdR red1-YFP-natR LEU2-CFP-mmi1*	5B, S7B	This study
JS157	*h90 ade6-M216 leu1 red1::bsdR dis3-mCherry-hphR*	5C, S7C	This study
JS158	*h90 ade6-M216 leu1 red1::bsdR rrp4-mCherry-hphR*	5C, S7C	This study
JS177	*h90 ade6-M216 leu1 ura4-D18 red1::bsdR mmi1::kanR mei4::ura4+*	4C, S6D	This study
JS180	*h90 ade6-M216 leu1 mtl1-cs5-YFP-kanR rrp6-mCherry-hphR*	6A	This study
JS182	*h90 ade6-M216 leu1 mtl1-cs5-YFP-kanR*	6B, S8	This study
JT719	*h90 ade6-M216 leu1 rrp6-mCherry-hphR*	6A	Lab stock
JT731	*h90 ade6-M216 leu1 dis3-GFP-kanR*	1B, S1B	Lab stock
JT856	*h90 ade6-M216 leu1 rrp4-GFP-kanR*	1B, S1B	Lab stock
JV579	*h90 ade6-M216 leu1 mmi1-ts3-(3HA)-adhT<<kanR*	S5C, S5D	Ref. 3
JV832	*h90 ade6-M216 leu1 ura4-D18 red1::ura4+*	3A	Ref. 22
JV969	*h90 ade6-M216 leu1 iss10::kanR*	2E, S3A, S3B, S3D	Ref. 22
JY450	*h90 ade6-M216 leu1*	3A, S3C, S4A, S4B	Lab stock
