# Supplementary material for: Meiotic gene silencing complex MTREC/NURS recruits the nuclear exosome to YTH-RNA-binding protein Mmi1
Source: PLoS Genet. 2020 Feb 3;16(2):e1008598. doi: 10.1371/journal.pgen.1008598 (PMC7018101; doi:10.1371/journal.pgen.1008598)
Supplement: S2 Table — (PDF) [file pgen.1008598.s010.pdf]

S2 Table. Primers used in this study.

Primer name	Sequence
act1 F	TGAGGAGCACCCTTGCTTGT
act1 R	TCTTCTCACGGTTGGATTTGG
mei4 F	AACCAAATGCTGAAACTCAAGAA
mei4 R	CGTTGACGTTTTCATAAAGGCTA
ssm4 F	GTGCAAAATTGAAACTATCAAAACA
ssm4 R	TTGGTTCATTTCTGTAAGGTCGT
rec8 F	AACGAACCCAAAGCAGTTACTACTC
rec8 R	GATCCACAGAAGGTAGATTAAATGCA
spo5 F	GGTTCTAGCGAGTTAGGGCTTTC
spo5 R	CCTGTGCTGCTGTAGAATAAGTATTGT
cti6 PROMPT F	CATCGGAGAACCTGGTTTGT
cti6 PROMPT R	TGCATTCCCAAATCAAGTCA
rpl402 PROMPT F	GAAAGAGGAACAATGTTTAGAAGTCA
rpl402 PROMPT R	AAGCCCTACGATTGCTGTGT
