# Supplementary material for: Meiotic gene silencing complex MTREC/NURS recruits the nuclear exosome to YTH-RNA-binding protein Mmi1
Source: PLoS Genet. 2020 Feb 3;16(2):e1008598. doi: 10.1371/journal.pgen.1008598 (PMC7018101; doi:10.1371/journal.pgen.1008598)
Supplement: Attachment — Submitted filename: response.docx [file pgen.1008598.s012.docx]

Dear Dr. Mata:

Thank you for the careful review of our manuscript (PGENETICS-D-19-01079). I herewith resubmit our revised manuscript. Below are our point-by-point responses to the reviewers’ comments. In addition, we have changed the author list to indicate that the first two authors (Yuichi Shichino and Yoko Otsubo) contributed equally to this work, since Y. Otsubo performed most of the experiments toward this revision. We would like to thank you for giving us an opportunity to revise our manuscript. We also appreciate the reviewers for their constructive comments on our manuscript that, I believe, have improved it further. We hope that the revised manuscript is satisfactory and found suitable for publication in *Plos Genetics*.

Thank you for your kind attention and consideration.

Sincerely,

Akira Yamashita

National Institute for Basic Biology,

Nishigonaka 38, Myodaiji, Okazaki, Aichi 444-8585, Japan

E-mail: ymst@nibb.ac.jp

Tel: +81-564-55-7512

Fax: +81-564-55-7656

Reviewer #1:
Major points:
1) The study focuses on the colocalization of exosome, Red1, and Mmi1 into nuclear foci that are thought to be degradation centers. However, results in this study questioned the importance of Mmi1 nuclear foci for RNA elimination. Expression of Red1 deletion mutant 196-245 shows complete loss of Rrp6 accumulation in nuclear foci (6%; Fig. 2C). Yet, the Red1 deletion mutant 196-245 only shows 2.5-fold accumulation of mei4 and ssm5 mRNAs, whereas the complete deletion of Red1 (which also shows 3-6% Rrp6 in foci; Fig. 1) leads to 20- and 12-fold accumulations of mei4 and ssm5 mRNAs, respectively (Fig. S2D). These results indicate that the presence of the nuclear exosome to Mmi1 nuclear foci is not absolutely required for degradation of mei4 and ssm4 mRNAs. This is a key point that needs to be clarified.

Response: We would like to thank the reviewer to pointing out this. We agree with the reviewer, and accordingly, have added the sentences to clarify that the Mmi1 foci are not sole sites for RNA degradation (page 19, line 10). As mentioned in the revised manuscript, we consider that Mmi1-mediated RNA degradation also takes place on the loci encoding its targets because Mmi1, Red1 and Rrp6 localize there. However, we feel that determination of the sites of degradation might be beyond the scope of this article. We would like to clarify this aspect in future after performing relevant studies.

2) The group of Torben Jensen recently showed that the human Red1 homolog, ZFC3H1, is required for the retention of targeted polyadenylated RNAs into nuclear degradation foci (Cell Reports 2018, 23: 2199). An important question that needs to be addressed before publication of this work is whether Red1 is also required for retention of meiotic transcripts in Mmi1 foci. The authors should perform RNA FISH on either ssm4 or mei4 mRNAs in red1-null cells to determine whether these RNAs are still tethered to Mmi1 foci or are now exported to the cytoplasm. This is important to dissect Red1’s role in nuclear exosome recruitment and nuclear RNA retention.

Response: We have already reported that Red1 is dispensable for tethering of *mei4* and *ssm4* mRNAs to nuclear foci, while Mmi1 prevents their nuclear exports by performing RNA FISH experiments (Shichino *et al.*, eLife. 2018;7:e32155. doi: 10.7554/eLife.32155). We have added the following sentences in the revised manuscript: “Intriguingly, ZFC3H1 is required not only for RNA degradation but also retention of target transcripts to nuclear foci [39, 40]. We have demonstrated that Red1 is dispensable for tethering of meiotic transcripts to nuclear foci, while Mmi1 prevents nuclear export of its targets by sequestering them to foci even when RNA degradation is dampened [33]” (page 20, line 1 from the bottom).

3) There are major inconsistencies in the RT-qPCR analysis of RNA levels presented throughout this study. For instance, whereas the mei4 mRNA is upregulated 20-fold in red1-null cells in Fig. S2D, it only shows 3-fold accumulation in Fig. 3B in the same deletion. The same is true for ssm4: 12-fold accumulation in red1 mutant in Fig. S2D, but 4-fold in Fig. 3B. The cit6 PROMT is up 15-fold in the red1 mutant in Fig. S2E, but shows 5-fold accumulation in the same mutant in Fig. S2F. This needs to be addressed.

Response: We apologize that we did not provide sufficient explanation of this point. As the reviewer pointed out, meiotic transcripts accumulated at higher levels in Figure 2E and S3B in the revised manuscript than in Figure 4A and S6A. We consider that this discrepancy may be caused by the difference of culture conditions used in the experiments. In Figure 2E and S3B, rich medium was used, while in Figure 4A and S6A cells were cultured in minimal medium to induce expression of the chimeric protein from plasmids. We have added the sentences to explain this (page 13, line 3 from the bottom; page 14, line 4). The reason why expression levels of *cti6* PROMT (and *rpl402* PROMPT) differ between Figure S3C and D in the revised manuscript might be caused by the use of the *red1-YFP* strain as wild-type cells in S3D, which does not affect significantly expression levels of meiotic transcripts (Figure 2E and S3B versus S3C).

4) Fig. 3A and S3 report physical association between Rrp6 and Mmi1. Because Mmi1 is an RNA-binding protein, it is very important to address whether the copurification of Rrp6 and Mmi1 is mediated by protein-protein interactions or depends on RNA by performing the coIP with and without benzonase. Alternatively, they could use RNA-binding defective mutants of Mmi1 such as Y352F or Y466F.

Response: As per the reviewer’s suggestion, we have added co-immunoprecipitation assay with RNase treatment (page 12, line 2 from the bottom, Figure S4B). The interaction between Rrp6 and Mmi1 was not affected by RNase treatment, indicating no significant role of RNA in the interaction.

5) The authors compare the molecular phenotypes of various Rrp6-YFP-Mmi1 fusions: full-length (FL), delta YTH, and delta SID. The delta SID mutant shows defective localization to nuclear foci and is unable to rescue RNA silencing in the red1 mutant. Accordingly, it is very important to show that this mutant (delta SID) is expressed at levels similar to the FL and delta YTH by Western blotting.

Response: According to the reviewer’s suggestion, we have added western blot analysis (page 15, line 1 from the bottom, Figure S6C). Expression of Rrp6-YFP-Mmi1-∆SID was rather increased compared to that of Rrp6-YFP-full-length Mmi1 and -∆YTH. This suggests that defects in the function and localization of Rrp6-YFP-Mmi1-∆SID are not caused by reduced expression of the chimeric protein.

6) It is not explained why analysis of Mmi1 function using the Rrp6-YFP-Mmi1 fusion was done in the mmi1-ts3 mutant and not in the deletion mutant, as done in Fig. 1 and Fig. S2. The use of the mmi1 deletion mutant represents a cleaner genetic system than the ts mutant.

Response: In the experiments in Figure S5C and D, we used minimal medium that allows expression of chimeric proteins from plasmids carrying an inducible promoter, while rich medium was used in Figure 1 and S3C. The deletion of *mmi1* leads to growth retardation in minimal medium even when combined with the *mei4* deletion. In Figure S5C and D, we examined whether chimeric proteins maintain the Mmi1 function, and thus we chose the temperature sensitive *mmi1* mutant for ease of use. In Figure 4C and S6D, we used the *red1∆* *mmi1∆* *mei4∆* to completely remove endogenous Mmi1. We have added the following sentences to explain this: “In these experiments, cells were cultured in minimal medium to induce expression of the chimeric protein. Since *mmi1∆* cells show growth retardation in minimal medium even when combined with the *mei4* deletion, we used the temperature-sensitive *mmi1* mutant instead of *mmi1∆*.” (page 13, line 3 from the bottom).

Minor points:
1) p. 6, lines 5-7: work by the Leatherwood (Chen et al. 2011 PLoS ONE) and Bachand (St-André et al. 2010, JBC) labs should be cited for studies on Pab2 and meiotic RNA elimination.

Response: According to the reviewer’s suggestion, we have added the citation (page 6, line 7).

2) p. 5, lines 5-8: the Mmi1 binding motif was also uncovered by a CRAC analysis of Mmi1 from the Vasiljeva lab (Cell Rep, 2015). Should cite.

Response: According to the reviewer’s suggestion, we have added the citation (page 5, line 8).

3) In Fig. 2A, the authors could show the various Red1 deletions using schematic. This would ease comprehension by readers.

Response: According to the reviewer’s suggestion, we have added a diagram of Red1 truncation series (Figure 2A).

Reviewer #2:
Major points:
1) An important aspect of the proposed model is that Red1 physically links the exosome to Mmi1 to promote efficient meiotic mRNAs degradation. Is this function specific to Red1 or dependent on MTREC? It would be informative to analyze a mutant of the core MTREC component Mtl1 in exosome foci formation and in meiotic mRNAs degradation in the presence of the chimeric Mmi1-Rrp6 construct.

Response: We appreciate the reviewer’s comment. Accordingly, we observed the localization of Rrp6 in *mtl1* mutant cells (*mtl1-cs5*) and found that Mtl1 plays a significant role in Rrp6 foci formation (Figure 6A and S8A). We also examined whether ectopic accumulation of meiotic transcripts in *mtl1-cs5* was suppressed by Rrp6-GFP-Mmi1. We showed that the chimeric protein suppressed aberrant expression of meiotic transcripts in *mtl1-cs5* as efficiently as wild-type Mtl1 (Figure 6B and S8B). These observations strongly suggest that the MTREC/NURS complex, rather than Red1 alone, connects Rrp6 with Mmi1.

2) The red1(∆196-245) mutant is defective for the formation of exosome and its own foci (Figure 2). This might suggest that the interaction between Mmi1 and the exosome is lost in this context. Another possibility is that Red1(∆196-245) is defective for its association with Mmi1, Rrp6 and/or Mtl1. Biochemical evidence should be provided.

Response: According to the reviewer’s suggestion, we have added co-immunoprecipitation assay in *red1(∆196-245)* cells (Figure 3B). We showed that the interaction of Red1(∆196-245) with Mmi1 was partially impaired, resulting in the weakened interaction between Rrp6 and Mmi1. We consider that this weakened but still significant association is consistent with the marginal effect of the residue 196-245 deletion on meiotic transcript elimination as pointed out by the reviewers.

3) The red1(∆196-245) mutant only marginally impacts meiotic mRNA degradation (Figure S2), suggesting that exosome foci formation may not be a critical determinant for transcripts degradation. The authors should discuss these apparent discrepancies in their model.

Response: We thank the reviewer for this insightful comment. As mentioned in our response to Reviewer #1 (Major point 1), we consider that Mmi1-mediated RNA degradation occurs not only at nuclear foci but also at other sites. The loci encoding Mmi1 targets are likely to be the site of RNA degradation, since it has been demonstrated by ChIP experiments that Mmi1, Red1 and Rrp6 also localize there. We have added the sentences to discuss this (page 19, line 10).

4) Data presented in Figure 1A/S1A indicate that Mmi1 is only partially involved in Rrp6 foci formation, contrary to Red1. This indicates that the exosome forms foci independently of Mmi1 and further questions the correlation between exosome foci formation and meiotic mRNA degradation.

Response: As the reviewer pointed out, deletion of *mmi1* has only partial impact on Rrp6 foci formation compared to *red1* deletion. We consider that Mmi1 and Red1 might function differently in Rrp6 foci formation, and that the latter contributes greatly than the former. We have modified the text based on this: “Cells lacking *mmi1* exhibited only a modest reduction in the frequency of Rrp6 foci formation and the number of foci per cell, indicating weak contribution of Mmi1 in Rrp6 foci formation. Foci formation of Mmi1 and Red1 was not interdependent (Fig 1A and S1A) [22, 33], and they might play divergent roles in Rrp6 foci formation.” (page 9, line 1).

5) The authors show that Rrp6-GFP-Mmi1-YTH∆ suppresses meiotic mRNA accumulation in red1∆ but not red1∆ mei4∆ mmi1∆ cells, suggesting that Rrp6-GFP-Mmi1-YTH∆ requires endogenous Mmi1. The authors should verify that endogenous Mmi1 associate with their chimeric construct in a SID-dependent manner. Also, one prediction is that the Mmi1 cofactor Erh1 should be required for the chimeric construct to function.

Response: As per the reviewer’s suggestion, we performed co-immunoprecipitation assay and confirmed the SID-dependent interaction between Rrp6-GFP-Mmi1 and endogenous Mmi1 (Figure S6E), supporting our hypothesis.

Minor points:
1) A quantification of the number of exosome nuclear foci in wt and mutant cells would be informative, especially since mei4∆ mmi1∆ cells appear to have less dots.

Response: According to the reviewer’s suggestion, we quantified frequencies of cells containing 0, 1, 2, 3, or 4 and more foci in Figure 1, 2, 4, 5 and 6. There was no significant difference in the distribution of the number of Rrp6 foci between wild-type and *mmi1∆* cells. We found that *red1* deletion affect the number of Pla1 and Pab2 foci (page 9, line 5 from the bottom, Figure 1C and D).

2) From the data presented (1B, S1B), there are very few Dis3 and Rrp4 dots outside the nucleolus. The percentage of foci frequency is not supported by the data. Could it be that Rrp6 is tethered to foci independently of the core exosome?

Response: We have added quantification of frequencies of cells containing 0, 1, 2, 3, or 4 and more Dis3 and Rrp6 foci (Figure 1B), as mentioned in our response to Minor point 1. The percentages of wild-type cells containing Dis3 or Rrp4 foci were lower than that of cells with Rrp6 foci, while no significant difference was observed in the distribution of the number of foci per cell. Dis3-GFP and Rrp4-GFP had strong fluorescent signal in the nucleolus. This might cause difficulties in observation of Dis3 and Rrp6 foci compared to that of Rrp6. We have added the following sentence in the revised manuscript: “Since the fluorescence signal of Dis3 and Rrp4 in the nucleolus in wild-type cells was more intense than that of Rrp6, the frequency of foci formation in or on the border of the nucleolus, and in nucleoplasm, might be underestimated.” (page 9, line 8).

3) Data in Figure 2B indicate that Red1 signals for the ∆2-347, ∆196-347 and ∆196-245 mutants are less intense. The authors should verify Red1 expression levels to exclude the possibility that that the lower frequency of dots is due to lower protein amount in the mutants.

Response: According to the reviewer’s suggestion, we confirmed that expression levels were similar among the Red1 deletion series (page 10, line 5, Figure S2A).

4) Expression analyses of additional CUTs should be provided to extend the results described for the cti6 PROMPT.

Response: We examined expression of *rpl402* PROMPT, which showed similar expression pattern to *cti6* PROMPT (Figure S3C, D and S6A, D). We tested several other CUT candidates, but their distinct accumulation was not observed even in *red1∆* of *rrp6∆* cells, at least in our experimental conditions.

Reviewer #3:
1) Throughout the manuscript, authors refer to the previously published work to support that red1 interact with rrp6 and mmi1 directly (for example page 12, 1st paragraph). The Co-IP of native complexes can not be used as an evidence for direct interaction. The current study does not provide this evince either. The proper biochemical experiments demonstrating direct interaction for recombinant proteins would benefit the manuscript.

Response: We appreciate the reviewer’s comment, and accordingly, performed *in vitro* binding assay (page 14, line 3 from the bottom, Figure 4B). We showed that Mmi1 directly interacted with Rrp6. We further demonstrated that this rather weak direct interaction was significantly enhanced by Red1. We believe that these observations strengthen our conclusion that the primary function of Red1 is to link Mmi1 to Rrp6.

2) The importance of the nuclear foci for degradation of meiotic transcripts is not clear to me from the data presented. Data presented in Figure S2D does not show accumulation of meiotic transcripts in red1 delta 196-245 where foci are not formed.

Response: We appreciate the reviewer’s comment. As mentioned in our response to Reviewer #1 (Major point 1) and Reviewer #2 (Major point 3), we have added the sentences to claim that nuclear foci are not sole sites for Mmi1-mediated RNA degradation (page 19, line 10). We assume that the genetic loci encoding Mmi1 targets are the site of RNA degradation. The fact that Mmi1, Red1 and Rrp6 accumulate at the loci supports this. It remains to be elucidated where Mmi1-mediated RNA degradation occurs. We will address this question and be able to clarify this in future.

3) In Figure S4E expression of rrp6 and mmi1 separately rescues red1 delta growth defect equally well to the expression of the Rrp6-Mmi1 fusion protein suggesting that while having more Rrp6 and Mmi1 can rescue the phenotype physical link between these may not be important.

Response: At this point, the reason why *red1∆* cells show growth retardation is not understood. Deletion of *mei4*, a crucial target of Mmi1, does not suppress the growth defect (Sugiyama *et al.*, Molecular Cell, 2016; Shichino *et al.,* eLife 2018), indicating that this defect is caused independently of Mmi1-mediated RNA degradation. Although the mechanism underlying suppression of the growth retardation by Rrp6 and Mmi1 remains unknown, we consider that it might occur independently of regulation of meiotic gene expression. We have added the following sentence to clarify this point; “We found that the slight growth retardation of *red1∆* cells at 25 to 36˚C, which has been shown previously [21, 22], was suppressed by expression of Rrp6, Mmi1 or Rrp6-GFP-Mmi1 as well as Red1, although the underlying mechanism remains unclear” (page 14, line 10).

4) Data in Fig 3B are quite central to the manuscript as they are meant to demonstrate that physical link between Rrp6 and Mmi1 is important for elimination of meiotic transcripts. However, the differences shown in this panel are not very striking, the numbers greatly varied between experiments (barely 3 fold difference for mei4 RNA in red1 delta in fig 3B compared to more than 20 fold difference in Figure S2D) and analyses is limited to only a couple of meiotic transcripts. I suggest extending analyses to more meiotic RNAs and presenting Northern blots and their quantifications.

Response: We are grateful to the reviewer for pointing this out. As mentioned in our response to Reviewer #1 (Major point 3), in Figure 4A in the revised manuscript, cells were cultured in minimal medium to induce expression of the chimeric proteins from plasmids, while we used rich medium in Figure 2E that enables rigorous repression of meiotic gene expression in wild-type cells. We thus consider that the less accumulation of meiotic transcripts in Figure 4A is due to the difference of culture conditions. We have added the sentences to explain this (page 13, line 3 from the bottom; page 14, line 4). As per the reviewer’s suggestion, we also examined the expression of *rec8* and *spo5*, both of which are known targets of Mmi1 (Figure S3B, C, S6A, D and S8B).

5) Section on self interaction of Mmi1 being important for degradation is the weakest part of the manuscript. I am sure that the manuscript benefit from this section at all. All the conclusions drawn based on indirect evidence and not convincing.

Response: We thank the reviewer for raising this point. As mentioned in our response to Reviewer #2 (Major point 5), we confirmed the SID-dependent interaction between Rrp6-GFP-Mmi1 and endogenous Mmi1 by co-immunoprecipitation (Figure S6E). We believe that these observations support the importance of self-interaction of Mmi1 for meiotic transcript elimination.

**Additional change:**

Yoko Otsubo has been changed to an author contributed equally to this work as the first author for having performed most of the experiments toward this revision.
