# Supplementary material for: Meiotic gene silencing complex MTREC/NURS recruits the nuclear exosome to YTH-RNA-binding protein Mmi1
Source: PLoS Genet. 2020 Feb 3;16(2):e1008598. doi: 10.1371/journal.pgen.1008598 (PMC7018101; doi:10.1371/journal.pgen.1008598)
Supplement: Attachment — Submitted filename: response.docx [file pgen.1008598.s013.docx]

Dear Juan:

Thank you for your kind handling our manuscript. We have modified the title, the abstract and the author summary according to the reviewer’s suggestion. We have also replaced Figure 6A with representative images consistent to the quantification. Below are our responses to the specific comments of the reviewer.

Many thanks again for your attention and consideration.

Sincerely,

Akira Yamashita

National Institute for Basic Biology,

Nishigonaka 38, Myodaiji, Okazaki, Aichi 444-8585, Japan

E-mail: ymst@nibb.ac.jp

Tel: +81-564-55-7512

Fax: +81-564-55-7656

Reviewer #2:
1) From data shown in Figure 6A and S8A, no Rrp6 foci are visible in the mtl1-cs5 mutant while the authors claim that there is only a mild effect on their formation. Images where such dots are present should be shown, consistent with the quantification.

According to the reviewer’s suggestion, we have replaced Figure 6A with representative images corresponding to quantitative data. We have also removed images of the nuclear region, because magnified cropped images are not appropriate for showing quantitative data.

2) The authors now show that Mtl1 is required for exosome foci formation and that the chimeric Mm1-GFP-Rrrp6 protein suppresses ectopic expression of meiotic mRNAs in the mtl1-cs5 mutant. Since there is no in vitro evidence that recombinant Mtl1 associates or not with Mmi1 and Rrp6, the authors should tune down the emphasis on Red1 in the title and the abstract, as they did in the discussion (“Our observations also imply that the MTREC/NURS complex, rather than Red1 alone, may link Mmi1 to the exosome”).

We appreciate the reviewer’s comment, and accordingly, have modified the title to clarify that the MTREC/NURS complex, not Red1 alone, links Mmi1 to the exosome as follows: “Meiotic gene silencing complex MTREC/NURS recruits the nuclear exosome to YTH-RNA-binding protein Mmi1”. We have also modified the abstract and the author summary.
